# Increasing the X-ray Diffraction Power of Protein Crystals by Dehydration: The Case of Bovine Serum Albumin and a Survey of Literature Data

**DOI:** 10.3390/ijms13033782

**Published:** 2012-03-21

**Authors:** Irene Russo Krauss, Filomena Sica, Carlo Andrea Mattia, Antonello Merlino

**Affiliations:** 1 Department of Chemical Sciences, University of Naples Federico II, Complesso Universitario di Monte Sant’Angelo, Via Cinthia, Naples I-80126, Italy; E-Mails: irene.russokrauss@unina.it (I.R.K.); filosica@unina.it (F.S.); 2 Institute of Biostructures and Bioimages, CNR, Via Mezzocannone 16, Naples I-80134, Italy; 3 Department of Pharmaceutical and Biomedical Sciences, University of Salerno, Via Ponte Don Melillo, I-84084 Fisciano, Italy; E-Mail: mattia@unisa.it

**Keywords:** serum albumin, protein crystallization, crystal dehydration, crystal quality, X-ray crystallography, post-crystallization treatment

## Abstract

Serum albumin is one of the most widely studied proteins. It is the most abundant protein in plasma with a typical concentration of 5 g/100 mL and the principal transporter of fatty acids in plasma. While the crystal structures of human serum albumin (HSA) free and in complex with fatty acids, hemin, and local anesthetics have been characterized, no crystallographic models are available on bovine serum albumin (BSA), presumably because of the poor diffraction power of existing hexagonal BSA crystals. Here, the crystallization and diffraction data of a new BSA crystal form, obtained by the hanging drop method using MPEG 5K as precipitating agent, are presented. The crystals belong to space group *C*2, with unit-cell parameters *a* = 216.45 Å, *b* = 44.72 Å, *c* = 140.18 Å, *β* = 114.5°. Dehydration was found to increase the diffraction limit of BSA crystals from ~8 Å to 3.2 Å, probably by improving the packing of protein molecules in the crystal lattice. These results, together with a survey of more than 60 successful cases of protein crystal dehydration, confirm that it can be a useful procedure to be used in initial screening as a method of improving the diffraction limits of existing crystals.

## 1. Introduction

Serum albumins are the major soluble protein constituents of the circulatory system and have many physiological functions [1–3]. The most important property of this group of proteins is to serve as transporters for a variety of endogenous and exogenous compounds including metabolites, drugs and other biologically active substances, mostly through the formation of non-covalent complexes at specific binding sites [2]. Various investigations have studied the structure and properties of serum albumins and their interactions with small molecules or with other proteins [2,4,5]. Bovine serum albumin (BSA) is one of the most extensively studied of this group of proteins, particularly because of its structural homology with human serum albumin (HSA). BSA is also frequently used as a model system for physical chemistry studies, as it is an easily available, low cost, protein with unusual ligand-binding properties [5,6].

BSA is composed of a single chain of 583 amino acid residues including 35 cysteines (forming a total of 17 disulfide bridges), which confer a high stability to the protein. The molecular weight for BSA, calculated from different techniques, ranges from 66,411 to 66,700 Da and “the best value” in solution is 66,500 Da [1]. Its secondary structure is constituted by 67% α-helix and its isoelectric point (pI) is reported in a pH range of 4.8 to 5.6 [6–8]. The structure and properties of BSA in solution are characterized by a versatile conformation that is a function of pH, ionic strength, and the presence of ions [9].

The structure of BSA in aqueous solution has been extensively studied in the past by small-angle X-ray scattering [10], quasi-elastic light scattering [11], hydrodynamic techniques [12], neutron scattering [13] and ^1^H NMR [14], but surprisingly its X-ray structure has not yet been solved. The main reason for this failure is that BSA crystals obtained up to now diffract to low resolution (the best diffraction obtained so far is 8 Å resolution) [15–17].

Here we describe the crystallization and preliminary X-ray diffraction studies of a new crystal form of BSA with two molecules in the asymmetric unit. We found that dehydration significantly improves the X-ray diffraction quality of these crystals. Dehydration is a post-crystallization treatment that tries to overcome the problems of loose packing of molecules and large solvent content, which are typical of protein crystals and lead to low-resolution diffraction. This procedure has previously been reported to increase the diffraction limit of many protein crystals. For a comprehensive survey of dehydration protocols the reader is referred to specific reviews which address this topic [18,19]. In this article, we also include a careful literature search of examples of improvements in X-ray diffraction properties of protein crystals, in an attempt to draw some conclusion from this review.

## 2. Results and Discussion

### 2.1. Crystallization of BSA

In the past, BSA crystals have been grown by a vapor diffusion technique from 50 mM potassium phosphate buffer, pH 6.2, 52% saturated ammonium sulphate at 298 K [15–17]. However, these crystals, which belong to space group P6 with unit cell parameters *a* = *b* =148.24 Å, *c* = 356.70 Å and *α* = 90°, *β* = 90°, *γ* = 120°, only diffract at low resolution (8–10 Å) [15,16].

**Figure 1:**
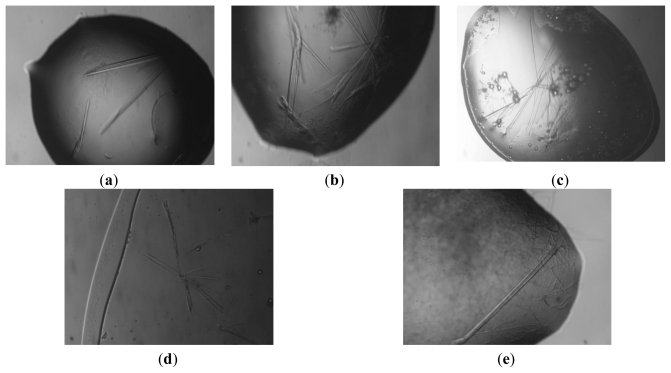
Image of typical bovine serum albumin (BSA) crystals grown by vapour diffusion (**a**–**e**). Crystals obtained from a crystallization solution containing 22–24% *w/v* MPEG 5K, 0.2M MgCl_2_, 0.1 M Tris HCl pH 7.8 (**a**–**c**) and 8 (**d–e**) and protein concentration of 20.0 mg mL^−1^.

Screening using polyethylene glycol of different molecular weights (2000–20,000 Da) as precipitating agent revealed new conditions for the crystallization of BSA. In particular, thin, small and fragile crystals appeared within 7 days using 30 mg mL^−1^ protein concentration with the hanging-drop method from crystallization conditions in which the reservoir solution contained 24% *w*/*v* MPEG 2K, 0.1 M Tris HCl pH 8. The quality of the crystals was improved by fine-tuning the concentration of protein (10.0–60.0 mg mL^−1^), changing the precipitants and their concentration, and evaluating the effect of divalent cations, such as CaCl_2_, ZnCl_2_, MgCl_2_. The best crystals (Figure 1a–e) were obtained from a crystallization solution containing 22–24% *w*/*v* MPEG 5K, 0.2M MgCl_2_, 0.1 M Tris HCl pH 7.8, 8.0 and 8.2 and BSA at 20.0 mg mL^−1^. Further optimizations of the crystallization conditions to grow larger and thicker crystals suitable for diffraction data collection at high resolution, using other methods (sitting drops or microbatch without oil [20]) failed.

Various cryosolutions (20% *v/v* glycerol, 300 mg mL^−1^ trehalose, 300 mg mL^−1^ saccharose) were prepared to examine their ability to cryoprotect the BSA crystals. Preliminary X-ray diffraction data collected at 100 K showed that even the best crystals (Figure 1a,b) were intrinsically disordered and that the largest ones diffracted at most to 8 Å resolution using glycerol as cryoprotectant. Application of an annealing protocol failed to improve the crystal diffraction quality. The latter method transiently returns the flash-cooled crystal to ambient temperature and has been shown to improve poor resolution and mosaicity, presumably caused by incorrect flash-cooling [21,22]. However, as reported in other cases [18,19,23–26], we found an increase in the diffraction power of BSA crystals by dehydration. A number of different trials for dehydrating crystals have been described in the literature. A comprehensive survey of the successfully used dehydration procedures is reported in Table 1 [18,19,24–85]. The dehydration process has been applied with success to crystals of proteins of various molecular weights, protein-protein and protein-ligand complexes. The resolution of the diffraction data collected from dehydrated crystals ranges from 1.1 Å to 4.5–5 Å, with resolution improvements that in some cases have been >10 Å; while the solvent content values range from 23% to 85%, with a decrease upon dehydration that generally has been <10%. The values of relative humidity in equilibrium with the solutions of the examined systems range from 74.3% to 99.5%. As expected, the best improvements in the X-ray diffraction power of protein crystals have been observed when the dehydration process has been applied to crystals with the highest solvent contents. Notably, the analysis of the Table suggests that even small changes in solvent content and relative humidity can promote favorable lattice rearrangements that dramatically improve the diffraction properties of crystals, as recently suggested by Russi *et al*. [26]. These findings underline the importance of reproducible and controlled crystal dehydration, such as that which can be obtained using modern devices available at synchrotron beamlines [86–88]. The data also confirm that at the start of a dehydration experiment, the relative humidity in equilibrium with the mother liquor is very often close to 100%, in agreement with recent data [89].

Various dehydration protocols have been used. The dehydration process traditionally consists of equilibrating the protein crystals over a reservoir with a higher percentage of precipitant [24,28–35]. The hanging drop containing the crystals is then allowed to dehydrate for 12 h to 3 days. The simplest implementation involves dehydration by air [25,36–42]. Good results have been also obtained when protein crystals are mounted in a specific and adjustable stream of humidified gas, where it is possible to control the relative humidity [26,43–48,86–88]. Finally, crystal dehydration can also be performed by transferring the crystals into a dehydrating solution, which is the original mother liquor with a higher concentration of precipitant [24,27,50–70] or with a different dehydrating agent [49,71–85].

In the present case, common cryoprotectants, various salts (for example malonate) and different molecular-weight PEGs were tested as possible dehydration agents, but ultimately the most successful experiment was obtained when crystals which were grown in 22–24% *w/v* MPEG 5K, 0.2 M MgCl_2_, 0.1 M Tris HCl pH 7.8 were directly transferred to a solution containing 30% *w/v* PEG 8K, 0.1M MgCl_2_, 0.05 M Tris HCl pH 7.8. Crystals did not show any signs of cracking during dehydration. After dehydration and cryocooling, the diffraction resolution of the crystals on the in-house X-ray equipment improved to 3.24 Å resolution. The diffraction resolution could be even further improved with a synchrotron radiation source. Assuming the presence of two BSA molecules in the asymmetric unit, the crystal volume per unit molecular weight (*V*_M_) is 2.3 Å^3^ Da^−1^, with a solvent content of 47%, which is within the normal range for protein crystals [92]. The solvent content of the crystals was reduced by 3–6% by dehydration. This process also produces a change in their relative humidity from 99.2% to 98.5%.

The application of molecular replacement, as detailed in the Experimental Section, enabled the identification of orientation and position of the two molecules in the asymmetric unit that gave a satisfactory fit to the experimental data. Refinement of the model, obtained by molecular replacement using phases derived from the structure of HSA is in progress.

The structural determination will provide a molecular basis for explaining numerous physical phenomena and for future docking and molecular dynamics studies on BSA complexes with drugs and other bioactive small molecules.

## 3. Experimental Section

### 3.1. Crystallization of BSA

Bovine serum albumin fraction V and all other reagents were purchased from Sigma Chemical Co. and used as supplied without further purification. BSA (80 mg/mL) was dissolved in 10 mM Tris-HCl buffer, pH 7.8. The protein concentration was determined spectrophotometrically using the extinction coefficient of 36,500 M^−1^ cm^−1^ at 280 nm [93].

Crystallization trials were performed at 293 K by the hanging-drop or sitting drop vapor-diffusion methods with 0.5 μL of protein and 0.5 μL of precipitant solution and a reservoir volume of 500 μL or using the microbatch without oil method [20] with the same volumes. Initial screens have included systematic PEG/pH and PEG/Ion screens. In particular, we prepared solutions with a formulation similar to the commercially available kits of Hampton Research. More than 100 different conditions were examined. In these crystallization experiments we varied the concentration of PEG from 10% *w/v* to 30% *w/v*, the molecular weight of PEG from 2000 Da to 20,000 Da and the pH from 7 to 8. The effect of divalent cations, such as CaCl_2_, ZnCl_2_, MgCl_2_ was also evaluated.

Needle crystals were obtained within 7 days from drops containing BSA (30 mg mL^−1^ in 10 mM Tris-HCl, pH 7.4) 24% *w/v* MPEG 2K and 0.1 M Tris HCl pH 8. An improvement in the quality of crystals was obtained using different salts and precipitant agents. In particular, well shaped crystals were grown using 22% *w/v* MPEG 5K, 0.2 M MgCl_2_, 0.1 M Tris HCl pH 7.8 as a precipitant solution. These crystals diffracted to 8 Å resolution. In all the experiments, standard 24-well linbro plates (Hampton Research, Laguna Niguel, USA) were used.

### 3.2. Dehydration

A significant improvement in the crystal diffraction quality was obtained by dehydration with PEG 8K. In this procedure, protein crystals were transferred in a loop to a 5 μL solution containing 30% *w/v* PEG 8K, 0.05 M Tris HCl pH 7.8 and 0.1 M MgCl_2_ for 10 min in the open air. After dehydration, the crystals were cryoprotected by soaking for 5–10 s in a solution consisting of 30% *w/v* PEG 8K, 0.05 M Tris HCl pH 7.8 and 0.1 M MgCl_2_, 20% *v/v* glycerol and tested for diffraction quality as above.

### 3.3. Data collection and Processing

X-ray diffraction data (3.24 Å resolution) were collected at the Institute of Biostructures and Bioimages (Naples, Italy), at 100 K using a Rigaku MicroMax-007 HF generator producing Cu *K*α radiation and equipped with a Saturn944 CCD detector. An oscillation range of 0.5° and an exposure time of 55 s were adopted for the experiments. The data sets were indexed, processed and scaled using the *HKL*-2000 package (Table 2) [94].

The overall *R*_merge_ was high at 15.4% and the *R*_merge_ value in the highest resolution bin was 31.9%. We attribute the high *R*_merge_ value as being primarily due to the large number of weak reflections that were measured and maybe to some radiation damage.

### 3.4. Structure Determination

The structure of the protein was solved by molecular replacement using the program Phaser [95] and HSA as search model (PDB code 2AO6 [96]). Water molecules were removed from the model prior to structure factor and phase calculations. The solution had an *R*-factor of 0.39.

## 4. Conclusions

For a long time the X-ray structure determination of BSA has been prevented due to the low diffraction power of its crystals. In this study, new BSA crystals were grown, X-ray diffraction data collected and the phase problem solved. BSA crystals that were initially unacceptable for structural analysis improved in diffraction limit by a process of dehydration. The best BSA crystals diffracted X-rays to a maximum resolution of 3.24 Å. Our results will be useful for numerous scientists who study the interactions of serum albumin with ligands, a field of interest for a great variety of biological, pharmaceutical, toxicological and cosmetic systems.

Our findings and previous literature results collected in Table 1 [18,19,24–85] confirm recent ideas that post-crystallization treatments can significantly improve X-ray diffraction protein crystal power. The analysis of the data does not enable us to define either a more promising dehydrating procedure or a more effective dehydrating agent. Rather, the review suggests that different procedures have to be tried, as the effects depend on both the protein nature and the crystal packing. Despite the high number of positive results, the technique remains little used. The take-home message of this work is that dehydration is one of the procedures that should be included in initial screening as a method to improve or at least modify the diffraction properties of existing crystals.

## Figures and Tables

**Table 1:** Dehydration of protein crystals and effect on solvent content and diffraction resolution.

Protein crystal	Reference	Crystal precipitant^a^	Dehydrating agent	Dehydration treatment	Space group (SG)	Solvent content b before (%)	Solvent content b after (%)	RH before (%)	RH after (%)	Resolution before(Å)	Resoluti on after (Å)
BSA	This work	22% MPEG 5K	30% PEG 8K	Transfer to drop of dehydr soln, 10 min	C2	50–53	47	99.2	98.5	~8 e	3.2 e
DsbG	[24]	20% PEG 4K	30% PEG 4K	Transfer to drop of dehydr soln, hang over reservoir of dehydr soln, 12h	C2	~90	53	99.3	98.4	~10^e^	2.0 e 1.7 d
FAD-indep ALS	[28]	6–8% PEG 8K 6–9% EG	Ppt 30% PEG 600	Hang over same dehydr soln, 12 h+ cryocool	C2	NR	52	NC	NC	2.9 e	2.6 e
Xis–DNA^X1-X2^	[29]	30% PEG 4K	35–40% PEG 4K	Replacing both the well and hangdrop solutions with dehydr soln	P3_1_21 or P3_2_21	NR	59	98.4	96.9–97.7	10 d	2.6 d
Aldolase C	[30]	25% PEG 8K 4% glucose	25% PEG 8K 4% glucose	Replacing both the well and hangdrop solutions with dehydr soln	P1	NR	NR	NC	NC	NR	3.0 e
Aldolase B	[31]	1.8–2.2 M AS 2% diaminooctane	3.5 M AS	Replacing both the well and hangdrop solutions with dehydr soln	P2_1_2_1_2	NR	NR	91.2–93.0	85.3	NR	2.7 e
Tom20 receptor	[32]	15% PEG 6K	25% PEG 6K	Replacing both the well and hangdrop solutions with dehydr soln	C2	NR	NR	99.6	99.0	3–8 d	2.1 d
transamidosome	[33]	10% PEG 4K	30% PEG 400 10% PEG 4K	Replacing the reservoir solution with dehydr soln	P2_1_2_1_2_1_ to P2_1_ upon dehydr	NR	65	99.8	<97.1	4.0 d	3.0 d
*X (or ADRP) domain of a variant of feline coronavirus*	[34]	2.6–2.8 M AS	2.6–2.8 M AS 4–17% glycerol	Replacing the reservoir solution with dehydr soln 12h	P4_1_2_1_2	NR	78	NC	NC	4.5 e	3.1 d
SecDF	[35]	26% PEG 400	50% PEG 400	Replacing both the well and hanging-drop solutions with dehydr soln	P4_3_2_1_2	75	74	97.7	92.3	4.2 d	3.7 d
DsbC-DsbDα	[36]	25% MPEG 5K 5% glycerol	40% MPEG 5K 10% glycerol	Air dehydrate 30 min + cryocool	P4_3_2_1_2	55	41	NC	NC	7.0 e	3.8 e 2.3 d
Pyruvate Dehydrogenase	[37]	6% PEG 3K	Ppt 35% glycerol	Air dehydrate for 28 months, rehydrate in same soln, cryocool	R32	NR	73	99.9	90.5	7.0 d	4.2 d
*E. coli* YbgL	[25]	0.8M sodium citrate	Ppt 10% EG	Annealing+air dehydrate (2 h)	C2	NR	57	NC	NC	~12^e^	2.6 e 1.8 d
*E. coli* YggV	[25]	35% AS	37.5% AS 10% glycerol	Annealing+air dehydrate (30 min)	P4_3_2_1_2	NR	38	89.5	<88.6	~12^e^	2.6 e 2.0 d
3-Dehydro dehy	[25]	11% PEG 8K	Ppt 10% glycerol	Annealing+air dehydrate (15 min)	P2_1_	NR	88	99.8	<97.9	ND	3.0 d
*Rv2002* gene product	[38]	20% PEG 3K	Ppt 10% MPD	Anneal + air dehydrate, 5 h	P3_1_2 1	NR	35	NC	NC	2.1 d	1.8 d
Peptide deformylase	[39]	12% PEG 4K	20% PEG 4K 10% PEG 400	Anneal + air dehydrate, 30 min	P2_1_2_1_2_1_	NR	50	99.7	<99.3	2.0 d	1.8 d
CLC Cl channel	[40]	22–32% Jeffamine	Ppt	Incub. in cryst. drop (5 months)	P222	NR	NR	NC	NC	7.5 d	4.0 d
Cytochrome ba_3_ oxidase	[41]	14–16% PEG 2K	20% glycerol 20% EG	Incub. under oil 2–4 h/air exp. 10 min	P4_3_2_1_2	NR	62	99.6–99.5	<93.2	4.0 d	2.3 d
5-Aminolaevulinic acid dehydratase	[42]	0.7 M 1,6-hexanediol		Air dehydrate, 30 min	P 4_2_2_1_2	NR	41 or 61	NC	NC	NR	2.7 d
Pea chloroplast photosystem I	[26]	26% PEG 4K		Controlled relative humidity device	P2_1_	NR	NR	99	97	6.0 d	4.0 d
Phosphoglycerate kinase	[26]	26% PEG 4K		Controlled relative humidity device	P2_1_2_1_2	NR	NR	98.5	97.5	3.0 d	1.8 d
Thioredoxin	[43]	10% PEG 1000		Controlled relative humidity device	C222_1_	NR	NR	NR	NR	8.0 d	2.9 d
F1-ATPase	[44]	14% PEG 6K		Controlled relative humidity device	P2_1_2_1_2_1_	NR	Reduction of 22%	99	90	NR	1.9 d
Dipeptidyl peptidase IV	[45]	20–22% PEG 2K		Controlled relative humidity device	P1	NR	NR	96.5	86.5	~10 d	3.0 d
Human GzmB	[46]	36% PEG 8K		Controlled relative humidity device	P2_1_2_1_2_1_	NR	NR	90	85	NR	3.1 d
Tricorn Interacting Factor F3	[47]	18% PEG 2K		Controlled relative humidity device	P3_2_21	NR	NR	98	94	BD	2.3 d
pMHC complexed with GTSGSPIADK	[48]	1.2 M K_2_HPO_4_ 0.6 M NaH_2_PO_4_		Controlled relative humidity device	C2	NR	70	94.5	93.5	~7 d	3.2 d
RFC–PCNA	[50]	15% PEG 3.4K	33% PEG 3.4K	Serial transfer into increasing PEG 3.4K, 2h	P2_1_2_1_2_1_	58	52	99.6	98.0	5.0 d	2.8 d
Penicillin G acylase	[51]	29% PEG 4K	36–70% PEG 4K 12–15% glycerol	Transfer to drop of dehydr soln (5–30 s)	P2_1_	NR	46	98.5	<84.1	8.0 e	2.2 e
Cytochrome ba_3_ oxidase mutants	[52]	6–7% PEG 2K	50% MPD, 14% PEG 2K	Transfer to drop of dehydr soln	P4_3_2_1_2 P4_1_2_1_2	NR	57–6	99.9	<99.6	2.6–3.0 d	2.3–2.4 d
ApoA-IV	[53]	22–28% PEG 3.4K	60% PEG 3.4K	Transfer to drop of dehydr soln, 12h	P6	64	59	99.3–98.6	90.8	3.5 d	2.7 d
Plant photosystem I	[54]	0.5% PEG 400 3–5% PEG 6K	0.5% PEG 400 40% PEG 6K	Transfer to drop of dehydr soln, 1 week	P2_1_	NR	NR	99.9	97.0	4.4 d	3.4 d
Nectin-1-EC complex	[55]	5% PEG 300	25% PEG 300	Transfer in var. steps to drop of dehydr soln	P2_1_3	NR	NR	99.6	97.4	~5 d	2.8 d
NgR	[56]	3.7 M NaCl	4.5 M NaCl	Transfer to drop of dehydr soln	P3_1_21	90	85	87.0	84.3	~5 d	3.2 d
Munc18c–syntaxin 41–29 complex	[57]	10–13% PEG 3.4K	25–30% PEG 3.4K	Transfer in var. steps to drop of dehydr soln	P2_1_3	54	53	99.8–99.7	98.9–98.4	4.3 e	3.7 e
HIV-RT:inhibitor	[58]	6% PEG 3.4K	46% PEG 3.4K	Serial transfer, 5% increments, 3 days	P2_1_2_1_2_1_	56	48	99.9	95.5	3.7 e	2.2 e
Pp 1,2-CCD	[59]	14% PEG 8K	16–18% PEG 8K 20 % glycerol	Transfer to drop of dehydr soln, 30–60s	*P*6_1_22	NR	63	99.7	<95.3	8–10 d	~3.3 d
*ec*SecA	[60]	6–9% PEG 35K	2 M KCl	NR	P2_1_	65	56	NC	NC	~3.5 d	2.0 d
MTCP-1	[61]	1.5 M AS	2.0 M AS	Soaked for 1–5 months	*P*6222	41	37	94.2	92.1	3.0 e	2.0 e
Trehalose phosphorylase	[27]	10% PEG 4K	18% PEG 4K	Various procedures	P2_1_2_1_2_1_	NR	60	99.8	99.5	~7–8 d	~3–4 d
Glutaryl-7-aminocephalosporanic acid acylase	[62]	4% PEG 8K 10–20% PEG 4K	30% PEG 8K 20% glycerol	Transfer to drop of dehydr soln	P2_1_2_1_2_1_	NR	NR	NC	NC	~4 d	1.6 e
EIICGlc(1–412, K394A, M17T, K150E)	[63]	32–35% PEG 400	>80% PEG 400	Transfer to drop of dehydr soln, 48 h.	P2_1_2_1_2_1_	NR	85	96.8–96.2	74.3	~8 d	4.5 e
MaoC-like dehydratase	[64]	5% PEG 6K	12% PEG 6K	Transfer to drop of dehydr soln, 30 min	P2_1_2_1_2_1_	NR	NR	99.9	99.8	ND	1.9 d
Fatty acid synthase	[65]	4–5% PEG 6K	23% PEG 6K	Transfer to drop of dehydr soln,	P2_1_2_1_2_1_ to P2_1_ upon dehydr	67	65	99.9	99.2	~8 d	~5 d
Nur	[66]	5% PEG 6K, 5% MPD	15% PEG 6K, 10% MPD	Transfer to drop of dehydr soln, 20 min	P3_1_	NR	65	99.9	<99.6	NR	2.4 d
Monoclinic lysozyme	[67]	10% NaCl	Satd NaCl solution	Transfer to drop of dehydr soln, 20 min	P2_1_	29	23	91.1	79.3	1.4 e	1.1 e
His6-RepE–DNA1	[68]	10% PEG 4K	12% PEG 4K	Transfer to drop of dehydr soln, 36 h	P2_1_	NR	63	99.8	99.8	~8 d	3.1 d
Ferredoxin reductase	[69]	16–18% PEG 10K	20% PEG 4K	Transfer to drop of dehydr soln, 15min	P3_2_2_1_1	NR	53	99.6–99.5	99.3	NR	2.2 d
MHC HLA-DQ2 complexed with gliadin peptides	[70]	25% PEG 4K	30% PEG 4K	dehydrated in a capillary containing dehydr soln, 3 days	I23	NR	40	98.9	98.4	~9 d	3.9 e
HCMV protease	[71]	16% PEG 4K	30% PEG 4K 0.15 M Na_2_SO_4_	Serial increase in reservoir conc, 3–5 days	P4_1_2_1_2_1_	58	56	99.6	<98.4	3.0 e	2.5 e 2.0 d
Human STAT1	[72]	10–12% PEG 400	10.5% PEG 400 10–30% PEG 4K	Transfer in var. steps to drop of dehydr soln	P6_1_22	NR	60	NC	NC	3.7 e	3.0 e
Monoclinic lysozyme	[73]	3% NaNO_3_	Satd K_2_CrO_4_ solution	Seal crystal in capillary, add plug of dehydr soln, for 15–20 h	P2_1_	33	22	NC	NC	2.5 e	1.7 e
Tetragonal lysozyme	[74]	0.48–0.75 M NaCl	Satd salt solutions	Seal crystal in capillary, add plug of dehydr soln, for days to weeks	P4_3_2_1_2	NR	NR	98.3–97.3	79.3	3.7 d	1.6 d
MmeI in complex with DNA	[75]	10% PEG 8K	20% PEG 4K	Changing the mother liquor for crystal growth	P1	NR	NR	99.8	99.3	~4 d	2.6 d
XRCC4–XLF complex	[76]	1.8 M TC	2.5 M AS	Transfer to 2.5 M AS 1 week + over 4 M AS, 5 days + 0.5 mM TB and 60% PEG 8000, 3 h	C2	NR	NR	NC	NC	~20 d	3.9 d
lipase–foldase complex	[77]	12% PEG 4K	30% PEG 8K	Transfer in var. steps to drop of dehydr soln	P3_1_21	62	60	99.8	98.5	~15 d	2.9 d
F1-ATPase	[78]	20% PEG 6K	20% PEG 6K 20% PEG 400	Serial transfer into dehydr soln	P2_1_2_1_2_1_	NR	62	NC	NC	6–8 d	3.1 d
EF-Tu-Ts	[79]	20% PEG 4K	28%–40%, var PEGs	Serial transfer, 5 min each	P2_1_2_1_2_1_	61	55	NC	NC	4.0 e	2.7 e
NF-κB P52-DNA	[80]	4–6% PEG 4K	Ppt 30% PEG 400 HA	Serial transfer into dehydr soln	I2_1_2_1_2_1_	52	49	NC	NC	3.5 d	2.0 d
CBL1	[81]	25% PEG 3.4K	7% MPEG 2K 0.7 M Li_2_SO_4_	Transfer to dehydr soln, 5 min	P2_1_2_1_2	NR	54	NC	NC	NR	2.9 d
Cx26	[82]	16–18% PEG	200 25–30%	TEG Serial transfer into increasing TEG, 1–2days	C2	NR	NR	NC	NC	~7 d	3.5 d
Nacetylglucosamine-1-phosphate Uridyltransferase	[83]	1.8 M AS	2.0 M AS Na malonate 5% glycerol	Serial transfer into dehydr soln	I432	Very high solvent content	82	93.0	<92. 1	3.8 e	3.4 e
SeMet YidC	[84]	22% PEG 3350 10% EG	30% PEG 3.4K 5–15% PEG 400	Serial transfer into dehydr soln	C2	50	47	NC	NC	3.5 e	1.8 e
DENV 3 RdRp	[85]	0.5% MPEG 5K	Var dehydr soln *i.e.*, 30% PEG 4K	Var procedures	C222_1_	NR	59	NC	NC	~20 d	1.8 d

AS, ammonium sulphate, BD, bad diffraction; Dehydr soln, dehydrating solution; EG, ethylene glycol; hang drop, hanging drop; HA, heavy atom;MPD, 2-methyl-2,4-pentanediol; MPEG, PEG monomethylether; ND, no diffraction, NR, not reported; PEG, polyethylene glycol; ppt, precipitant; satd, saturated; TC, triammonium citrate, TB, tantalum bromide; TEG, triethylene glycol; var, various.

a Crystal precipitant information does not include details of buffers and other additives used in crystallization;

b Solvent content was not always reported by authors. In some cases it has been calculated from information provided in the text of the paper;

c Relative humidity (RH) values have been calculated using the online calculator available at http://go.esrf.eu/RH, as described by Bowler and co-workers [89]. Concentrations have been converted from *w/v* to *w/w* using: *w/w* = *w/v* density^−1^, where density values are taken from literature [90,91];

d X-ray diffraction resolution at a synchrotron source;

e X-ray diffraction resolution on a rotating anode source.

**Table 2:** Data collection statistics.

Space group	*C*2
Cell parameters	
*a* (Å)	216.45
*b* (Å)	44.72
*c* (Å)	140.18
*β* (°)	114.5
Resolution limits (Å)	50.00–3.24
Highest resolution shell (Å)	3.32–3.24
No. of observations	57717
No. of unique reflections	18006
Completeness (%)	88.8 (81.5)
I/*σ* (I)	5.5 (2.9)
Average multiplicity	3.2 (2.4)
*R*_merge_ (%)	15.4 (31.9)
Mosaicity	1.2

Note: Values in parentheses correspond to the highest resolution shell.
